# *MicroRNA-139*, an Emerging Gate-Keeper in Various Types of Cancer

**DOI:** 10.3390/cells11050769

**Published:** 2022-02-22

**Authors:** Christiaan J. Stavast, Iris van Zuijen, Stefan J. Erkeland

**Affiliations:** Department of Immunology, Erasmus MC University Medical Center, 3015 GD Rotterdam, The Netherlands; c.stavast@erasmusmc.nl (C.J.S.); i.vanzuijen@erasmusmc.nl (I.v.Z.)

**Keywords:** *MIR139*, cancer, tumor suppressor, transcriptional regulation, microRNAs

## Abstract

Mounting data show that *MIR139* is commonly silenced in solid cancer and hematological malignancies. *MIR139* acts as a critical tumor suppressor by tuning the cellular response to different types of stress, including DNA damage, and by repressing oncogenic signaling pathways. Recently, novel insights into the mechanism of *MIR139* silencing in tumor cells have been described. These include epigenetic silencing, inhibition of POL-II transcriptional activity on gene regulatory elements, enhanced expression of competing RNAs and post-transcriptional regulation by the microprocessor complex. Some of these *MIR139*-silencing mechanisms have been demonstrated in different types of cancer, suggesting that these are more general oncogenic events. Reactivation of *MIR139* expression in tumor cells causes inhibition of tumor cell expansion and induction of cell death by the repression of oncogenic mRNA targets. In this review, we discuss the different aspects of *MIR139* as a tumor suppressor gene and give an overview on different transcriptional mechanisms regulating *MIR139* in oncogenic stress and across different types of cancer. The novel insights into the expression regulation and the tumor-suppressing activities of *MIR139* may pave the way to new treatment options for cancer.

## 1. Introduction

MiRNAs are small non-coding RNAs (19–22 nt) and post-transcriptionally regulate the expression of target mRNAs involved in cell proliferation and differentiation, stress responses and the prevention of oncogenesis [1,2,3]. Almost all primary miRNA transcripts (pri-miRNAs) are transcribed by RNA Polymerase II and commonly contain a 5′ cap and, in most cases, a poly-A tail [4] (for reviews, see [5,6,7,8]). The pri-miRNAs form a hairpin structure and are cleaved by the RNase III enzyme DROSHA that is bound to RNA binding protein DiGeorge syndrome chromosome region 8 (DGCR8) into a premature miRNA (pre-miRNA) [9,10,11]. The pre-miRNA is transported to the cytoplasm by Exportin-5 (XPO5), where the hairpin is cleaved by the RNase III enzyme DICER and stabilized by trans-activation-responsive RNA binding protein (TRBP) [12,13,14,15,16]. Only one of the two miRNA strands, either miRNA-5p or miRNA-3p, is then loaded by the RNA-induced silencing complex (RISC) loading complex (RLC) into RISC [17,18,19,20,21], which consists of Argonaute 2 (AGO2), DICER, TRBP, proteins of the Glycine-Tryptophan protein of 182 kDa (GW182) family, such as trinucleotide repeat-containing gene 6A–6C (TNRC6A–TNRC6C), and the carbon catabolite repressor 4-negative on TATA (CCR4-NOT) complex [22,23,24,25,26,27,28]. Nucleotides 2–7 at the 5′ end of the miRNA, the seed region, are critical for target binding specificity on the 3′ UTR of target transcripts [7,23,29]. The activities of miRNAs are highly cell-type- and cellular-state-dependent [30,31]. There is strong evidence for miRNA functions in stress responses [3]. *MIR139* is an example of a stress-responsive gene. Silencing of *MIR139* is critical for the oncogenic transformation of cells. The role for *miR-139* in the diagnosis and prognosis of cancer is described in the following reviews [32,33]. Here, we discuss the recent findings regarding the transcriptional and post-transcriptional regulation of *MIR139* in cellular stress conditions. Furthermore, we discuss some of the major oncogenic targets that are regulated by *miR-139*.

## 2. Genomic Localization and Host Gene PDE2A

*MIR139*, encoding *miR-139-5p* and *miR-139-3p*, is a well-conserved miRNA located on human chromosome 11q13.4 in intron-1 of the Phosphodiesterase 2A (*PDE2A*) gene. PDE2A is an essential cAMP-cGMP hydrolyzing enzyme and is a signal transducer in different cellular processes [34,35,36]. Genomic deletion of *Pde2a* in mice (B6; 129P2-Pde2A< tm1Dgen>/H; EM: 02366) is embryonically lethal and mutant mice die in utero at embryonic day (E) 15.5 [37]. In addition, despite multiple attempts of our research team, mice with a genomic deletion of the putative *Pde2a* promoter could not be generated, indicating that *Pde2a* is essential for survival [38]. In agreement, other investigators have found that *Pde2a* KO embryos display lethal defects in fetal liver development and hematopoiesis [39]. Livers from *Pde2a* KO embryos (E14.5) displayed an increased level of cleaved Caspase-3, expressed decreased levels of anti-apoptotic protein BCL2 and contained Annexin-V-positive apoptotic cells compared to heterozygous and wild-type (WT) littermates [39]. In addition, in *Pde2a*-deficient fetal livers, the development of myeloid and erythroid lineages is impaired [39]. However, the *Pde2a* deficiency does not affect the colony-forming capacity of myeloid progenitors, indicating that PDE2A is dispensable for the expansion and maturation of hematopoietic progenitors. 

**Figure 1:**
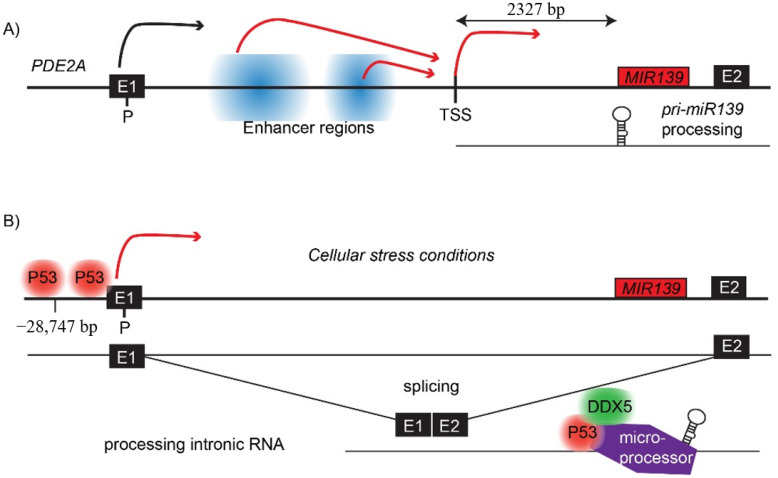
Overview of the transcriptional regulation of *MIR139*. (**A**) Schematic representation of the PDE2A locus (chr11: 72,605,000–72,644,500) with the promoter (P), transcriptional start site of *MIR139* (TSS), the first two exons of *PDE2A* (E1 and E2), enhancer regions (in blue) and *MIR139* (red box). The transcription of *PDE2A* is indicated by the black arrow. The enhancer regions are critical for *MIR139* transcription (red arrows). (**B**) Schematic overview of the model in which, under cellular stress conditions, p53 binds to the *PDE2A* promoter and stimulates transcription (red arrow) and processing of *pri-miR-139*.

Despite a significant correlation between the expression of *PDE2A* and *MIR139* in lung cancer cell lines [40], we and other investigators have shown that *miR-139-5p* and/or *miR-139-3p* expression is not correlated to *PDE2A* expression levels in leukemia [38], gastric [41] and colorectal cancer cells [42]. We have recently shown that *MIR139* expression is strongly silenced in MLL-AF9 AML, whereas the expression of *Pde2a* was not affected [38]. These data indicate that post-transcriptional mechanisms other than splicing and subsequent processing of *pri-miR-139* by the microprocessor complex play a role in the stability and processing of *miR-139-5p* and *miR-139-3p*. In agreement, a transcriptional start site (TSS) of *MIR139* was established on *pri-miR-139* by rapid amplification of cDNA ends (5′-RACE) in gastric SGC-7901 cells, which is 2327 base pairs upstream of the *pre-miR-139* (Figure 1A) [41]. Together, these data indicate that *MIR139* is regulated, at least in part, independently of *PDE2A*. The different mechanisms involved in transcriptional and post-transcriptional *MIR139* regulation are discussed below. An overview of the different in vitro and in vivo models used to study *MIR139* in cancer can be found in Table 1.

## 3. *MIR139* Is Induced by p53-Mediated Cellular Stress Response

The level of *miR-139-3p*, but not *miR-139-5p*, is elevated in hematopoietic stem and progenitor cells (HSPCs) of Fanconi anemia patients and in HSPCs of nucleotide excision repair gene *Ercc1*-deficient mice [45]. Elevated *miR-139* levels in these cells are a direct result of interstrand DNA crosslinks (ICLs) and cause apoptosis [45]. In agreement, treatment of normal HSPCs with ICL-inducing agent Mitomycin C induces *miR-139-3p* expression. This effect is counteracted by increased *miR-199* expression in these cells [45]. Blocking of *miR-139-3p* with antagomirs rescues HSPC expansion in colony assays, demonstrating the relevance of *miR-139-3p* for ICL-mediated bone marrow failure. The expression of *miR-139-3p* is undetectable in Fanconi AML cells, suggesting that *MIR139* silencing is an oncogenic driver event that allows for the acceptance of high oncogenic stress levels in the affected cells, which ultimately leads to the transformation of pre-leukemic Fanconi myeloid progenitor cells towards AML [45]. We showed that p53 is responsible for ICL-induced bone marrow failure and that loss of p53 drives leukemogenesis in this model [45]. The loss of p53 coincided with the loss of *miR-139* expression in *Ercc1*-deficient leukemia cells. This result suggests that *MIR139* expression is regulated by the tumor suppressor p53.

A well-conserved p53-responsive element (*p53RE*) is mapped at the −28,747 bp position downstream of *MIR139* (Figure 1B), which was experimentally confirmed by ChIP experiments with human lung carcinoma cells after p53 induction [52]. Treatment of lung cancer cell lines with Inauhzin-C, a p53-activating compound, induces *MIR139* expression only in p53-positive cells, but not in p53 KO cells [52]. In the human colon cell line HT29-tsp53 expressing a temperature-sensitive variant of murine p53 (V135A), *miR-139-5p* and *miR-139-3p* were both rapidly upregulated at the permissive temperature, as well as the expression of *PDE2A* [53,54]. P53 binds to the promoter of *PDE2A* (Figure 1B), which may explain the correlation of *miR-139* levels with the increased expression of the PDE2A gene. A different study of colorectal cancer presents evidence for differential transcriptional regulation of *pri-miR-139* transcripts independent of *PDE2A* [79]. However, it is likely that one of the mechanisms by which p53 regulates *MIR139* expression is via the induction of *PDE2A* expression. *PDE2A* is already expressed in the absence of p53, which suggests that p53 may play a role in the processing of intronic *pri-miR-139* that occurs after splicing of *PDE2A* premature mRNA (Figure 1B). The tumor suppressor p53 has been shown to enhance miRNA biogenesis by association with DEAD-Box Helicase-5 (DDX5) in cellular stress responses [80]. Mechanisms other than p53-mediated transcription are involved the regulation of *MIR139* expression, which will be discussed in the next section.

## 4. *MIR139* Expression Is Repressed in Various Types of Cancer

### 4.1. The Expression of MIR139 Is Frequently Silenced in AML

Acute myeloid leukemia (AML) is a complex disorder of the bone marrow (BM) that results from the aberrant clonal expansion of myeloid progenitors that have acquired genomic aberrations and mutations, which provide a growth advantage and a block of differentiation [81]. In addition, miRNAs are aberrantly expressed in all subtypes of AML [82,83,84]. We [38,45] and other investigators [46,47,48,49,50,51] have found that *MIR139* is a tumor suppressor gene that is frequently silenced in leukemia, including Fanconi anemia-related leukemia, caused by interstrand crosslink (ICL)-induced DNA damage [45], Breakpoint Cluster Region Protein-Abelson Murine Leukemia Viral Oncogene Homolog 1(BCR-ABL)-mediated leukemogenesis [49], AML [46,47] and T-cell acute lymphoblastic leukemia [51]. We found that *miR-139* expression levels are low in normal HSPCs and induced by DNA damage [45]. We showed that *miR-139-3p* is not expressed in clinical AML samples. In agreement, analysis of deep sequencing data of AML samples from the Cancer Genome Atlas (TCGA) further indicated that *miR-139-3p* is not expressed or is expressed at low levels in AML [45]. In addition, *miR-139-5p* is undetectable in most AML cases, except for a low expression level in AML samples characterized by a M2 FAB classification and t(8;21), in samples with inv-(16) and some cases with various abnormalities (our unpublished data). Furthermore, *miR-139-5p* is downregulated in different subtypes of AML and in AML cell lines, compared to differentiated myeloid cells, which further supports a role of *miR-139-5p* as a tumor suppressor [46]. Krowiorz et al. show that *miR-139-5p* is downregulated in FLT-3 mutants, in NPM1/FLT3 double mutants and in CN AML compared to the average expression of all AML samples tested in the TCGA cohort [47]. In this comparison, *miR-139* expression in the t-(9;11) cases was very similar to other subtypes of AML. However, we presented strong evidence that *MIR139* is downregulated in AML expressing the MLL-AF9 oncogene compared to normal HSPCs. Together, these data indicate that the tumor suppressor gene *MIR139* is commonly silenced in AML.

### 4.2. The Effect of MIR139 KO on Development and Oncogenesis

To investigate the functions of *MIR139* in oncogenesis, we have generated *Mir139* knockout (KO) mice [38,43]. We found that C57BL/6J *Mir139* KO mice were born at Mendelian ratios, developed normally and had expected HSPC counts and mature hematopoietic cell types in peripheral blood and BM [38,43]. Notably, the expression of *Pde2a* was not affected in the HSPCs of *Mir139* KO mice [38]. A panel of 22 *Mir139* KO and 18 *Mir139* WT mice were monitored for the development of leukemia and other types of cancer for 2 years. Only one *Mir139* KO mouse developed acute leukemia at the age of 89 weeks, suggesting that additional oncogenic driver events are needed for oncogenesis (unpublished data). Clinical data show that acute myeloid leukemia (AML) patients with the lowest *miR-139* levels have a poor prognosis [46]. In agreement, we found that *Mir139* KO HSPCs gave rise to more and larger colonies when transformed with the MLL-AF9 oncogene in colony-forming unit assays, showing that *miR-139*-depleted leukemia cells have a growth advantage [38]. Whether *MIR139* silencing is a critical early driver of leukemogenesis still needs further investigation.

*Mir139* KO mice were also used for the investigation of *MIR139* tumor suppressor functions in different types of cancer. For instance, other investigators have found that *Mir139* KO mice are highly susceptible to the development of dextran sulfate salt (DSS)-induced colitis and colon cancer [44]. The investigators found that *miR-139-5p* expression is lost in colorectal cancer tissue over time. The proliferation rate of *Mir139* KO tumor cells was enhanced, confirming the growth advantage of *Mir139* KO tumor cells. Furthermore, Zhou et al. showed increased expression levels of anti-apoptotic genes *Bcl-Xl* and *Bcl-2* in *Mir139* KO cells compared to *Mir139* WT tumor cells in colitis-associated colorectal cancer [44]. They found that the expression of *Mir139* dampens the expression of phosphorylated MAPK, NF-κB and STAT3, all factors that drive inflammation and colitis-associated oncogenesis [44]. These data demonstrate that *MIR139* inactivation is an oncogenic driver event that results in prolonged intracellular stress-induced signaling and the survival of cells.

### 4.3. MIR139 Is Silenced by POLR2M Downstream of PRC2 in AML

**Figure 2:**
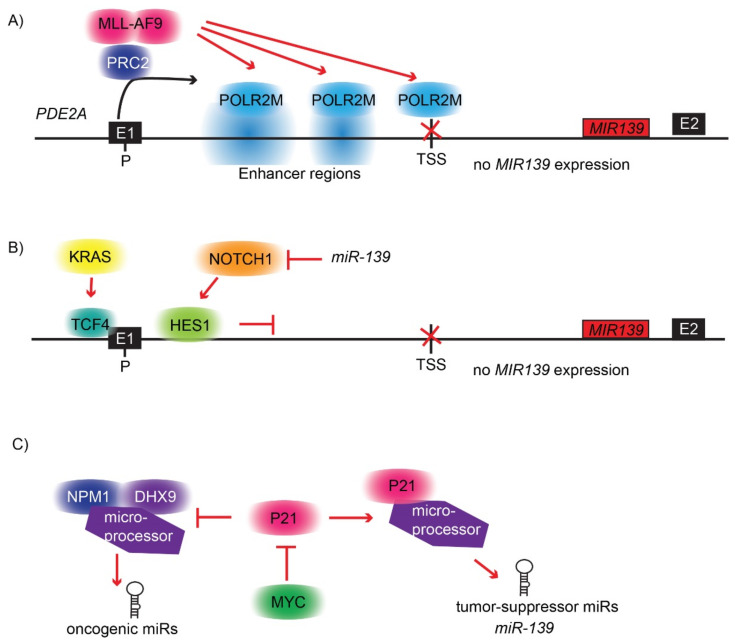
Overview of *MIR139* molecular silencing mechanisms. (**A**) Model of *MIR139*-silencing mechanism in AML. PRC2 is recruited to the promoter region of PDE2A downstream of MLL-AF9. The host gene PDE2A is expressed at normal levels (black arrow). However, under these conditions, POLR2M is recruited to the enhancer regions and to the TSS of *MIR139* (red arrows), which results in transcriptional silencing of *MIR139* (red cross). (**B**) Additional silencing mechanisms of *MIR139*. Mutant KRAS recruits TCF4-β-CATENIN to the TSS of *MIR139*, thereby inhibiting transcription. Activated NOTCH1 signaling results in HES1 binding close to the promoter of *PDE2A*, which causes downregulation of *MIR139* expression. *NOTCH1* is a validated target of *miR-139* (red inhibitor arrow), thereby creating a feed-forward loop. (**C**) P21 is a central player in the regulation of *pri-miR-139* processing. Activation of P21 stimulates the processing of tumor suppressor pri-miRNAs, including *pri-miR-139*. However, when P21 is repressed by the oncogene MYC (red inhibitor arrow), this results in further stimulation of the microprocessor that is bound by NPM1 and DHX9 to preferentially process oncogenic miRNAs. KRAS-induced MYC transcription activates KMAT expression, which stabilizes NPM1-DHX9 complex, thereby contributing to the enhanced processing of oncogenic miRNAs.

Polycomb group (PcG) proteins have been implicated in the silencing of tumor suppressor genes [85,86,87]. Mounting evidence shows that *MIR139* is silenced by the Polycomb repressive complex-2 (PRC2) in various types of cancer [38,70,73,74]. PRC2 consists of the methyltransferase Enhancer of Zeste Homolog-1/2 (EZH1/2), Embryonic Ectoderm Development (EED), Suppressor of Zeste 12 Homolog (SUZ12) and Retinoblastoma-binding protein-4 (RBBP4) [88,89]. EZH1/2 hypermethylates K27 on Histone-H3 (H3K27), which marks silenced genes [90]. Deregulation of PRC2 contributes to AML pathogenesis [91,92,93]. We have recently identified POLR2M as a novel downstream mediator of PRC2-induced transcriptional repression of *MIR139* by interaction with the TSS and enhancer regions of *MIR139* (Figure 2A) [38]. POLR2M (also known as GDOWN1) pauses POL-II-mediated transcription by binding to the POL-II complex [94,95]. Promoter-proximal pausing of POL-II at TSSs has been correlated with H3K27me3 and PcG-silenced genes [96,97]. We have shown that depletion of *POLR2M* results in the expression of *MIR139* and induction of apoptosis of human and mouse MLL-AF9 AML cells [38]. The repressive activity of POLR2M can be reversed by interaction with the multi-subunit protein complex Mediator, which results in the high induction of transcription [95]. Mediator is a transcriptional co-regulator that consists of approximately 30 subunits, including MED4, MED6, MED7, MED8, MED10, MED11, MED14, MED 17, MED21 and MED22, which are essential for Mediator function [98]. Various Mediator subunits are mutated, aberrantly expressed or deregulated in human cancer including leukemia [99,100]. For example, MED12 mutations are found in up to 9% of chronic lymphocytic leukemia (CLL) cases and contribute to the pathogenesis by activating NOTCH signaling [101,102]. CDK8 transiently associates with Mediator and controls its activity [98]. The Mediator complex provides communication between active enhancers and promoters by forming a molecular bridge within actively transcribed genes and interacts with transcription factors, POL-II and elongation factors [103,104,105,106,107]. In addition, Mediator binds to acetylated Histones [108]. For instance, H4K16 acetylation inhibits the interaction of MED5 and MED17 to chromatin [108,109]. Moreover, H3K27 acetylation correlates with high levels of Mediator complex subunits at regular and super enhancers with high POL-II occupancy [79,110,111]. Proteomics studies in yeast revealed that 17 subunits of the Mediator complex are dynamically phosphorylated by an unidentified kinase in response to stress and regulate the expression of stress-induced genes [112] (for review, see [98]). Vice versa, there is evidence that the phosphorylation of transcription factor ELK in response to ERK activation fine-tunes the interaction with Mediator and thereby transcriptional activity [113]. However, how Mediator interacts with other transcription factors to facilitate the transcription of *MIR139* remains elusive.

### 4.4. Oncogene Mediated MIR139 Silencing

Multiple well-known oncogenes silence the expression of *MIR139* in cancer. For instance, NOTCH1 signaling suppresses *MIR139* expression via the transcriptional repressor HES1, which binds to the E-box site at position +644 bp in the *PDE2A* gene in glioma cells (Figure 2B) [67]. In this study, the authors show that *miR-139* modulates stemness by inhibiting Wnt/β-catenin signaling, which is a hallmark of cancer [67]. As *NOTCH1* is a direct target of *miR-139* (discussed below), this creates a feedback mechanism that fine-tunes NOTCH1 signaling (Figure 2B).

In colorectal cancer, *MIR139* is strongly downregulated in KRAS mutant cells compared to KRASWT cells [55]. In this study, the investigators found that the expression of *MIR139* is controlled by two TCF4 sites flanking the TSS of *MIR139* (Figure 2B). TCF4 binds to β-catenin and transcriptionally silences target genes. Furthermore, the investigators found that, in KRAS mutant cells, the *MIR139* expression is suppressed in a WNT3A-activated β-catenin-TCF4 complex-dependent manner [55]. The expression of *MIR139* is inhibited in KRASWT-overexpressing lung tumor cells in a very different way [62]. KRAS overexpression induces the expression of the long non-coding RNA KRAS-Induced-Metastasis-Associated-Transcript 1 (*KMAT1*) by activation of MYC-mediated transcription. KMAT1 induces the processing of oncogenic miRNAs, including *miR-17*, *miR-18* and *miR-27*, through stabilization of the RNA-binding proteins DExH-Box Helicase 9 (DHX9) and Nucleophosmin-1 (NPM1). NPM1 binds to DHX9, which is part of the microprocessor complex, in a RNA-dependent manner and is involved in the selection of pri-miRNAs for processing [62]. On the other hand, MYC silences CDKN1A (P21), which is a component of the microprocessor complex, by interaction with DROSHA in specific conditions. The authors show that pri-miRNA transcripts of tumor-suppressing miRNAs, including *pri-miR-139*, are not processed when P21 is transcriptionally silenced by MYC [62]. When P21 is overexpressed, it antagonizes the stimulating effects of DHX9 and NPM1 on the biogenesis of oncogenic miRNAs, whereas the expression of a subset of tumor-suppressing miRNAs, including *pri-miR-139*, is enhanced. P21 interacts directly with the microprocessor complex and with a subset of pri-miRNAs. In addition, the authors showed that the levels of *pre-miR-139* and *miR-139* were both dependent on P21 expression, whereas the expression of *pri-miR-139* was not [62]. This indicates that P21 is involved in the selective processing of *pri-miR-139* by the microprocessor complex (Figure 2C). The abovementioned *MIR139* regulatory genes, including KRAS, MYC, NOTCH1 and NPM1, are frequently mutated in leukemia. However, whether the above-described aberrant *MIR139* mechanisms play a direct role in leukemogenesis is unknown and needs further investigation.

### 4.5. Post-Transcriptional Regulation of MIR139

*Mir139* expression is regulated by a post-transcriptional mechanism. The first indication for the post-transcriptional regulation of *pre-miR-139* was found in colorectal cancer samples from patients in which *miR-139* was detected at reduced levels, whereas the levels of *pre-miR-139* were similar to the expression in normal tissue [79]. These data suggest that DICER or specific RNA-binding proteins, which interact with *pre-miR-139* and regulate further processing, are deregulated in colorectal cancer. In addition, our data in MLL-AF9 leukemia, where *Pde2a* is normally expressed and spliced but *miR-139* levels are strongly decreased, can only be explained by reduced *pri-miR-139* stability and/or processing. The following mechanisms may explain this phenomenon. In MLL-AF9 leukemia, P21 is silenced by Inhibitor of DNA binding 1 (ID1), which is critical for MLL-AF9 leukemogenesis [56,114]. According to the role for P21 in the selection of *pre-miR-139* for further processing as described above, the downregulation of P21 in MLL-AF9 AML may largely explain the low mature *miR-139* levels, but this still needs proper validation.

We found that the enhancer regions in intron-1 of *Pde2a* and upstream of *Mir139* are critical for normal *Mir139* expression levels [38]. Thus, our results indicate that other still unknown mechanisms interact with the enhancer regions and are involved in *Mir139* expression regulation. DROSHA and DGCR8, associated with transcriptional regulators, are thought to be recruited co-transcriptionally and process pri-miRNAs during transcription [115]. Recent data present evidence that super enhancers boost the transcription and DROSHA/DGCR8-mediated processing of a subset of cell-specific miRNAs [116]. Whether these interactions exist at the enhancers of *MIR139* is currently under investigation.

Downregulation of *miR-139* activities in tumors may be due to the overexpression of competing target RNAs, the so-called sponge activity. For instance, LINC00324 overexpression acts as a *miR-139* sponge, thereby releasing Insulin-like Growth Factor-1 Receptor (*IGF1R*) from *miR-139* regulation and increasing the IGF1R protein expression in non-small-cell lung cancer [63]. In addition, the 3′-UTR of LNCRNA *PCAT6* competes with the 3′-UTR of *BRD4* transcripts for *miR-139* binding and downregulates *miR-139* expression when overexpressed in pituitary adenomas [76]. The levels of *miR-139* may also be downregulated by circular RNAs with *miR-139* sponge activities. To date, only a few circular RNAs have been reported in the regulation of *MIR139*. Strikingly, the circular RNAs that are described to have sponge activity against *miR-139* contain only one binding site for either *miR-139-5p* or *miR-139-3p*. To be able to compete with other mRNAs containing sites for *miR-139* in their 3′-UTR, the expression of functional circular RNAs should be at least higher than the target mRNA. For instance, *Circ-0038718* consists of exons 2 and 3 derived from the gene encoding the Interleukin-4 Receptor (*IL4R*) and is highly overexpressed in hepatocellular carcinoma [75]. *Circ-0038718* contains one *miR-139-3p* binding site and interacts with AGO2-loaded *miR-139-3p*, thereby competing for oncogenic *miR-139-3p* mRNA targets. Furthermore, *CircKIF4A* acts as a sponge for *miR-139-3p* in glioma, thereby activating oncogenic WNT3A signaling [68]. In addition, *Circ-0000218* controls *miR-139-3p* levels in a very similar way in laryngeal and colorectal cancer [57,77]. *CircBACH2* is a circular RNA that is expressed at elevated levels in papillary thyroid carcinoma and downregulates the expression of *miR-139-5p* [78]. How the interaction of AGO2-loaded *miR-139* with circular RNA causes degradation of the miRNA is unknown. However, mechanisms by which miRNA-target mRNAs degrade miRNAs have been described [117]. Furthermore, how the increased expression of non-coding RNA or circular RNA, containing only one interaction site, competes with all other *miR-139* targets in such a way that it efficiently represses the activity of *miR-139* on other target mRNAs is not well understood and suggests a specific RNA-mediated miRNA degradation pathway. An overview of *miR-139* regulators is given in Table 2.

## 5. *MIR139* Targets Involved in Oncogenesis

Overexpression of *MIR139* in Kasumi-1 and SKNO-1 cells, both AML cell lines with t-(8;21), and mRNA expression profiling revealed *EIF4G2* as one of the most downregulated transcripts [46]. According to Targetscan, the database that lists predicted miRNA targets [118], *EIF4G2* has one well-conserved 8-mer site for *miR-139-5p* in the 3′-UTR. This *miR-139*-mediated silencing of *EIF4G2* was confirmed on the protein level in Kasumi-1 cells [46]. ShRNA-mediated silencing, at least in part, phenocopied the effects of *miR-139* expression on the viability and proliferation of Kasumi-1 cells [46]. *EIF4G2* mRNA lacking *miR-139* binding sites in the 3′-UTR rescued the anti-proliferative and apoptotic effects of *miR-139* overexpression in Kasumi cells. We recently confirmed *Eif4g2* as a critical target of *miR-139* in mouse MLL-AF9 AML [38], suggesting that *Eif4g2* is a more common *miR-139* target in leukemia. In addition, EIF4G2 has been recently identified as a *miR-139* target in other types of cancer, including glioblastoma and colorectal cancer [119,120]. EIF4G2 is important for protein synthesis [121]. Accordingly, *MIR139* overexpression resulted in reduced overall protein expression, which may explain the inhibitory effects of *miR-139* expression on tumor cell proliferation and survival [46].

There is mounting evidence that *miR-139-5p* targets *NOTCH1* in different cell types, thereby preventing aberrant NOTCH1 signaling and oncogenic transformation. According to Targetscan, *NOTCH1* contains one broadly conserved site for *miR-139-5p* in the 3′-UTR. This explains that, in cancers with silenced *MIR139* expression, the expression of *NOTCH1* is increased. Forced expression of *miR-139-5p* causes the downregulation of *NOTCH1* via direct binding to the 3′-UTR in colorectal cancer and inhibits the migration and invasion of tumor cells [58,59]. Increased levels of RP11-59H7.3, a long non-coding RNA that is aberrantly expressed and correlates with poor prognosis of colorectal cancer, compete with *NOTCH1* for *miR-139* binding, thereby enhancing NOTCH1 oncogenic functions [60]. Another tumor-suppressing activity of *miR-139-5p* via *NOTCH1* repression in colorectal cancer is the prevention of CD44^+^/CD133^+^-associated multidrug resistance [61]. In ovarian cancer, overexpression of the lncRNA *SNHG3* competes for *miR-139-5p* binding, thereby increasing *NOTCH1* levels [71]. The authors showed that reduced *miR-139-5p* expression enhanced the proliferation and migration of ovarian cancer cells. In addition, downregulation of *Notch-1* expression and reduced blood glucose levels were observed as a result of oxidative-stress-induced *miR-139* expression in the liver cells of diabetic mice [66]. Furthermore, reduced *miR-139* levels as a consequence of chronic fine particulate matter (PM 2.5)-induced cellular damage in the lung cause *Notch-1* upregulation and Epithelial–Mesenchymal Transition (EMT) in mice [64]. Vey similar tumor-suppressing activities of *miR-139* on *NOTCH1* levels, tumor cell growth, EMT and metastasis have been described in a mouse model for glioma [69]. NOTCH1 signaling is frequently deregulated in various types of leukemia [122,123,124]. Whether *MIR139* plays a major role in NOTCH1 signaling during leukemia development remains to be investigated.

Other *miR-139* targets that are described in leukemia and some other types of cancer are *BTG3* [46], the RNA-binding protein *ELAVL1* [45,65,72], *Tetraspanin-3* (*TSPAN3*), *MAX Network Transcriptional Repressor* (MNT) [48], *15-Hydroxyprostaglandin Dehydrogenase* (*HPGD*) and *Protein Tyrosine phosphatase Receptor Type-T* (*PTPRT*) [38]. Although knockout and knockdown studies show the relevance of these downregulated targets for *miR-139*-mediated functions as a tumor suppressor, more in-depth studies are needed for the understanding of their oncogenic role in leukemia.

## 6. Conclusions

Mounting data show that the targeting of miRNAs and their controlled pathways may be a successful approach for anti-cancer treatment [82,125,126]. It becomes increasingly evident that *MIR139* is a critical tumor suppressor gene in different types of cancer and that the deregulation of *MIR139* transcription, processing or targeting activity inhibits its tumor-suppressive activities. Although the mechanism of *MIR139* silencing in various types of cancer is not fully unraveled, targeting *MIR139* to reactivate its expression is a promising avenue for novel targeted therapies.

## Figures and Tables

**Table 1:** Overview of experimental models used for functional *MIR139* investigation in the various types of cancer.

Experimental Model	Type of Cancer, Cell Types	References
*MIR139* KO mice	Bone marrow, T-cells and colon	[38,43,44]
*Ercc1* KO mice	Bone marrow, Fanconi anemia	[45]
Mouse 32D cells	AML	[45]
Mouse MLL-AF9 leukemia and human AML cell lines MOLM-13, THP-1, MV4-11, HL-60, HEL and U937, Kasumi-1, SKNO-1	MLL-AF9 AML, AML	[38,46]
Human AML and CML, AML cell lines NB4, HP-1, KG-1a, OCI-AML3, U937, HL-60 Human T-ALL cell lines HPB-ALL, TALL-1, KOPTK1, Jurkat, CCRF-CEM, Molt16	Diverse types of leukemia	[47,48,49,50,51]
Colon cancer cell lines HT29, SW480, SW620, KM12, SW116, HCT116, HCT-8, HCT-116, LoVo, Caco2, DLD1, LS180, NCM460, HcoEpic and Human colon cancer in NOD/SCID mice	Colon cancer	[42,52,53,54,55,56,57,58,59,60,61]
Human lung cancer cells, H460, IC11LC13, NSCLC cell lines A549, H1299, H1975, HCC827, H1650, H460, SK-MES-1 and SPC-A-1, PM2.5-treated mice	Lung cancer	[40,62,63,64,65]
Diabetes mouse model, Streptozotocin-injected Kunming mice	Blood cells, liver	[66]
Patient-derived glioma stem-like cells, Human glioma cell lines LN229, A172, SHG44, T98G, U87 and U251, BALB/c nude mice	Glioma	[67,68,69]
Primary Human Ovarian cancer, cell lines A2780, SKOV3, OVCAR3 and OV90	Ovarian cancer	[70,71,72]
SGC-7901, MKN-45 and AGS	Gastric cancer	[41]
SW1990, BxPC-3, PANC-1 and AsPC-1	Pancreatic cancer	[73]
HepG2, PLC/PRF/5, MHCC97L and SM. Human HCC cell lines (SK-Hep-3B, HepG2, HCC-LM3) and MHCC97-HMC-7721	Liver cancer	[74,75]
Cell lines RC-4B/C (CRL-1903) and GH3 (CCL-82.1)	Pituitary adenomas	[76]
Cell line SNU46	Laryngeal squamous cell carcinoma	[77]
Human cell lines K1, IHH-4, BCPAP and TCP1	Papillary thyroid carcinoma	[78]

**Table 2:** Overview of Activators and Repressors of *MIR139* Expression.

Regulator of *MIR139*	Activator/Repressor	Type of Cells	*miR-139* Targets	References
PDE2A (host gene)	Activator	Lung cancer cell lines		[40]
Epigenetic	Repressor	AML	*EIF4G2, BTG3*	[46]
PRC2	Repressor	AML	*EIF4G2*, *HPGD*, *PTPRT*	[38,70,73,74]
POLR2M	Repressor	AML	*EIF4G2, HPGD, PTPRT*	[38]
P53	Activator	HSPCs Fanconi anemia Colon cancer	*ELAVL1*	[45]
			*PDE4D*	[52]
			*P53 targets*	[53,54]
				
NOTCH1/HES1	Repressor	Glioma	Wnt/β-Catenin	[67]
TCF4	Repressor	Colorectal cancer	JUN, DVL1, CTNNB1, ZEB1	[55]
KRAS/MYC/P21	Repressor	Lung cancer		[62]
Competing RNAs:	Repressors			
*LINC00324*		Non-small-cell lung cancer	*IGF1R*	[63]
*PCAT6*		Pituitary adenomas	*BRD4*	[76]
*Circ-0038718*		Hepatocellular carcinoma		
*CircKIF4A*		Glioma	*WNT3A*	[68]
*Circ-0000218*		Laryngeal/Colorectal cancer	*RAB5A, RAB1A*	[57,77]
*CircBACH*		Papillary thyroid carcinoma	*LMO4*	[78]
*SNHG3*		Ovarian cancer	*NOTCH1*	[71]

## Data Availability

Not applicable.
